# Pharmacokinetics and UPLC-MS/MS of Delsoline in Mouse Whole Blood

**DOI:** 10.1155/2018/9412708

**Published:** 2018-10-11

**Authors:** Lingjiu Shao, Yue Jin, Huiyan Fu, Jianshe Ma, Xianqin Wang, Yongxi Jin, Congcong Wen

**Affiliations:** ^1^Department of Nephrology, Wenzhou Central Hospital, Wenzhou 325000, China; ^2^Analytical and Testing Center, School of Pharmaceutical Sciences, Wenzhou Medical University, Wenzhou 325035, China; ^3^Department of Rehabilitation, Wenzhou Municipal Hospital of Traditional Chinese Medicine, Wenzhou 325005, China; ^4^Laboratory Animal Centre, Wenzhou Medical University, Wenzhou 325035, China

## Abstract

Delsoline, a major alkaloid of *Delphinium anthriscifolium* Hance, has both a curare-like effect and a ganglion-blocking effect and is used to relieve muscle tension or hyperkinesia. A ultraperformance liquid chromatography-tandem mass spectrometry (UPLC-MS/MS) method was established for the determination of delsoline in mouse blood, and the pharmacokinetics of delsoline after intravenous administration (1 mg/kg) and intragastric administration (9, 6, and 3 mg/kg) were studied. Gelsenicine served as an internal standard, and a UPLC BEH C18 chromatographic column was used. The mobile phase consisted of acetonitrile and 0.1% formic acid; the gradient elution flow rate was 0.4 mL/min. The MRM model was used for the quantitative analysis of delsoline *m/z* 468.3⟶108.1 and the internal standard *m/z* 327.1⟶296.1. Mouse blood samples were treated with acetonitrile precipitation to remove proteins. In the concentration range of 0.1–1000 ng/mL, delsoline in mouse blood showed a good linearity (*r*^2^ > 0.995), and the lower limit of quantitation was 0.1 ng/mL. The intraday precision relative standard deviation (RSD) was below 14%, and the interday precision RSD was below 15%. The accuracy ranged between 94.3% and 110.1%, the average recovery was above 90.8%, and the matrix effect ranged between 97.0% and 102.5%. The UPLC-MS/MS method was sensitive, rapid, and selective in the study of pharmacokinetics of delsoline. The absolute bioavailability of delsoline was 20.9%.

## 1. Introduction

*Delphinium anthriscifolium* Hance, belonging to the genus *Delphinium* of family Ranunculaceae, is a perennial herb growing in the regions of Guangdong, Guangxi, Guizhou, Hunan, and Jiangxi of China [1, 2]. It has effects of dispelling wind and dampness, activating collaterals, and relieving pains and is used to treat rheumatism, hemiplegia, indigestion, and cough. It can also be used externally to treat carbuncle sores. *Delphinium anthriscifolium* Hance is composed of alkaloids, fixed oils, lipoids, and glycosides. Delsoline and eldeline are the two main alkaloids of *D. anthriscifolium* Hance [3–5]. Delsoline has a curare-like effect and a ganglion-blocking effect and can be used to relieve muscle tension or hyperkinesia. The analysis of delsoline pharmacokinetics will provide a theoretical basis for the study of its absorption and distribution in the body. To our knowledge, the pharmacokinetics and UPLC-MS/MS of delsoline have not been previously reported in the literature.

Compared with LC-MS/MS and micellar liquid chromatography, the UPLC-MS/MS method is more sensitive and better suited for the research of pharmacokinetics [6–11]. UPLC-MS/MS is a powerful segregation tool for the analysis of complicated Chinese traditional medicinal components and the in vivo metabolism of complex compounds.

In the current study, a UPLC-MS/MS method was established for the determination of delsoline in mouse blood and to study the pharmacokinetics of delsoline after intravenous administration (1 mg/kg) and intragastric administration (9, 6, and 3 mg/kg), and then, absolute bioavailability of delsoline was obtained.

## 2. Materials and Methods

### 2.1. Experimental Reagents

**Figure 1:**
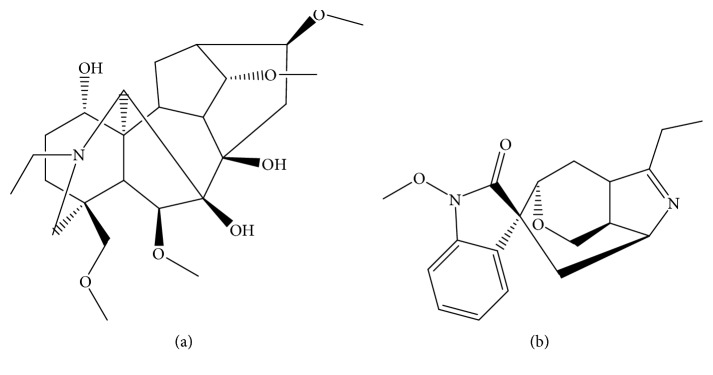
Chemical structures of delsoline (a) and gelsenicine (internal standard, (b)).

Delsoline (purity >98%; Figure 1(a)) and gelsenicine (internal standard, purity >98%; Figure 1(b)) were purchased from Chengdu Mansite Pharmaceutical Co., Ltd. (Chengdu, China). Chromatographically pure acetonitrile and methanol were purchased from Merck Ltd. (Darmstadt, Germany). Ultrapure water was prepared by the Millipore Milli-Q purification system (Bedford, MA, USA). ICR mice (body weight 20–22 g) were purchased from the Animal Experimental Center of Wenzhou Medical University (Wenzhou, China).

### 2.2. Experimental Instruments

ACQUITY I-Class UPLC and XEVO TQS-micro-Triple Quadrupole Mass Spectrometer (Waters Corp, Milford, MA, USA) was used. MassLynx 4.1 software (Waters Corp.) was used to collect data and control instruments.

The chromatographic column was UPLC BEH C18 (2.1 mm × 50 mm, 1.7 m), and the column temperature was set at 30°C. The mobile phase consisted of acetonitrile and 0.1% formic acid. Gradient elution was performed, the flow rate was 0.4 mL/min, and the elution time was 4 min. The elution procedure was 10% acetonitrile for 0–0.2 min, 10%–80% acetonitrile for 0.2–1.5 min, 80% acetonitrile for 1.5–2.0 min, 80%–10% acetonitrile for 2.0–2.5 min, and 10% acetonitrile 2.5–4.0 min.

Nitrogen was used as the desolvation gas (900 L/h) and the cone gas (50 L/h). The capillary voltage was set at 2.0 kV, the ion source and the desolvation temperatures were set at 150°C and 450°C, respectively. The MRM model was used for the quantitative analysis of delsoline *m/z* 468.3⟶108.1 and the internal standard *m/z* 327.1⟶296.1.

### 2.3. Preparation of Control Solution

Delsoline (1.0 mg/mL) and gelsenicine (1.0 mg/mL) stock solutions were prepared with methanol : water (50 : 50, v/v). Different concentrations of standard working solutions were prepared by diluting the delsoline stock solution with methanol. An acetonitrile solution containing 20 ng/mL internal standard gelsenicine was prepared by diluting gelsenicine stock solution with acetonitrile. All solutions were stored at 4°C.

### 2.4. Preparation of Standard Curve

The standard curve of blood delsoline was prepared by adding appropriate amounts of the standard working solution to blank mouse blood, and the concentrations of delsoline in the mouse blood were 0.1, 0.5, 2, 5, 20, 50, 200, 500, and 1000 ng/mL. The range of the standard curve was 0.1–1000 ng/mL. Quality control (QC) samples of four blood concentrations (0.1, 0.4, 180, and 900 ng/mL) were prepared in the same way as the standard curve.

### 2.5. Sample Processing

20 *μ*L blood sample and 100 *μ*L acetonitrile (containing 20 ng/mL internal standard gelsenicine) were added in a 1.5 mL Eppendorf tube, mixed using a vortex mixer for 1.0 min, and centrifuged at 13,000 rpm for 10 min at 4°C. 80 *μ*L of the supernatant was transferred into the liner tube of a vial, and 2 *μ*L was used for UPLC-MS/MS analysis.

### 2.6. Method Validation

The method validation was established according to the United States Food and Drug Administration (FDA) guideline of method validation for biologics [12]. The items of validation included selectivity, matrix effect, linearity, precision, accuracy, recovery rate, and stability.

#### 2.6.1. Selectivity

The selectivity of the method was evaluated by analyzing blank blood, blank blood spiked with delsoline and internal standard, and mouse blood samples.

#### 2.6.2. Linearity

Standard series of various concentrations were prepared with standard working solution, and the standard concentrations ranged from 0.1 to 1000 ng/mL. The peak area was measured under the same condition as the tested blood sample. A standard curve was plotted using the ratio of peak area/internal standard peak area against sample concentrations, and the linearity of the experiment was assessed by the standard curve.

#### 2.6.3. Precision and Accuracy

Precision and accuracy were assessed by six repeated measurements of the blood samples at the concentrations of 4 QC samples (0.1, 0.4, 180, and 900 ng/mL). Precision was expressed as relative standard deviation (RSD), and the intraday and interday precision were determined by measuring QC samples for three consecutive days. The degree of agreement between the mean value and the true value of the QC sample was measured for three consecutive days to determine the intraday and interday accuracy.

#### 2.6.4. Recovery Rate and Matrix Effect

The recovery rate was evaluated by comparing the peak area of QC samples with the corresponding standard peak area. The matrix effect was assessed by comparing the peak area of four concentrations (0.1, 0.4, 180, and 900 ng/mL) obtained by adding standard solution to blank blood after protein precipitation, with the peak area of standard solution diluted by acetonitrile-0.1% formic acid (1 : 1, v/v).

#### 2.6.5. Stability

The stability of delsoline in mouse blood was investigated by analyzing the mouse QC samples under three storage conditions. Stability, including stability in the vial, short-term stability (room temperature, 2 h), long-term stability (−20°C, 30 days), and freezing thawing stability (−20°C to room temperature), were assessed by comparing the peak area with freshly prepared standard samples.

### 2.7. Study of the Pharmacokinetics

Before the experiment, 1.0 mg/mL drug solution was freshly prepared by dissolving 4.0 mg delsoline in purified water containing 0.01% HCl. 24 mice were randomly divided into four groups with 6 mice in each group. One group underwent intravenous administration (1 mg/kg), and the other three groups underwent intragastric administration (3, 6, and 9 mg/kg). Venous blood was taken from the tail veins of mice at 0.0833, 0.5, 1, 2, 3, 4, 6, and 8 h after intravenous or intragastric administration of delsoline. Blood samples were cryopreserved in 1.5 mL Eppendorf tubes at −20°C.

The area under the blood concentration-time curve (AUC), mean residence time (MRT), blood clearance (CL), apparent volume of distribution (*V*), maximum blood concentration (*C*_max_), and half-life (*t*_1/2_) was analyzed. The pharmacokinetic parameters were fitted using noncompartmental models by DAS 2.0 software (China Pharmaceutical University). The equation for bioavailability is expressed as the following: absolute bioavailability = intragastric AUC/intravenous AUC × 100%.

## 3. Results and Discussion

### 3.1. Method Optimization

Selection of positive or negative electrospray ionization (ESI) is often discussed in methodological studies. Delsoline is an alkaloid and an alkaline compound. It is more suitable for the detection using positive ESI. Our data also verified that ESI in the positive ion mode was more sensitive than in the negative ion mode.

Liquid chromatography conditions separated the endogenous interfering substances from the analyte and internal standard retention times as much as possible, and the chromatographic column and mobile phase played a decisive role in the chromatographic behavior [13]. We tried the gradient elutions using acetonitrile-0.1% formic acid, acetonitrile-10 mmol/L ammonium acetate solution (containing 0.1% formic acid), methanol-0.1% formic acid, and methanol-10 mmol/L ammonium acetate solution (containing 0.1% formic acid). The most satisfactory shape of the chromatographic peak and retention time could be obtained with the elution using acetonitrile-0.1% formic acid. Therefore, BEH C18 (2.1 mm × 50 mm, 1.7 *μ*m) was used as the chromatographic column, and acetonitrile-0.1% formic acid was used as the mobile phase in this study.

Prior to the UPLC-MS/MS analysis, removal of proteins and potential interference is critical in the method establishment. We tried direct precipitation methods using methanol, acetonitrile, and methanol-acetonitrile (1 : 1, v/v) and found that the precipitation method using acetonitrile had the best effect. Considering that blood samples were more complicated than plasma components, the endogenous substances in 20 *μ*L blood were precipitated using 100 *μ*L acetonitrile. The direct precipitation method using acetonitrile was fast and simple to remove proteins and could provide a good recovery rate and an acceptable matrix effect. Therefore, the precipitation method using acetonitrile was used for the treatment of blood samples.

Selection of internal standard is also important in the establishment of method. In this study, gelsenicine, which has a similar structure to delsoline, was selected as an internal standard. Results showed that the chromatographic retention time and the process of ionization mass spectrometry were similar and met the requirements of internal standard for UPLC-MS/MS analysis.

Compared with the conventional HPLC analysis, UPLC-MS/MS is faster and more sensitive in the quantitative detection of delsoline in blood. It takes only 4 min to complete the analysis of blood samples by this method, which saves time and solvents. In addition, the LLOQ of delsoline is relatively low (0.1 ng/mL), which made this method capable to determine a relatively low blood concentration at the last time point of sampling.

### 3.2. Method Validation

**Figure 2:**
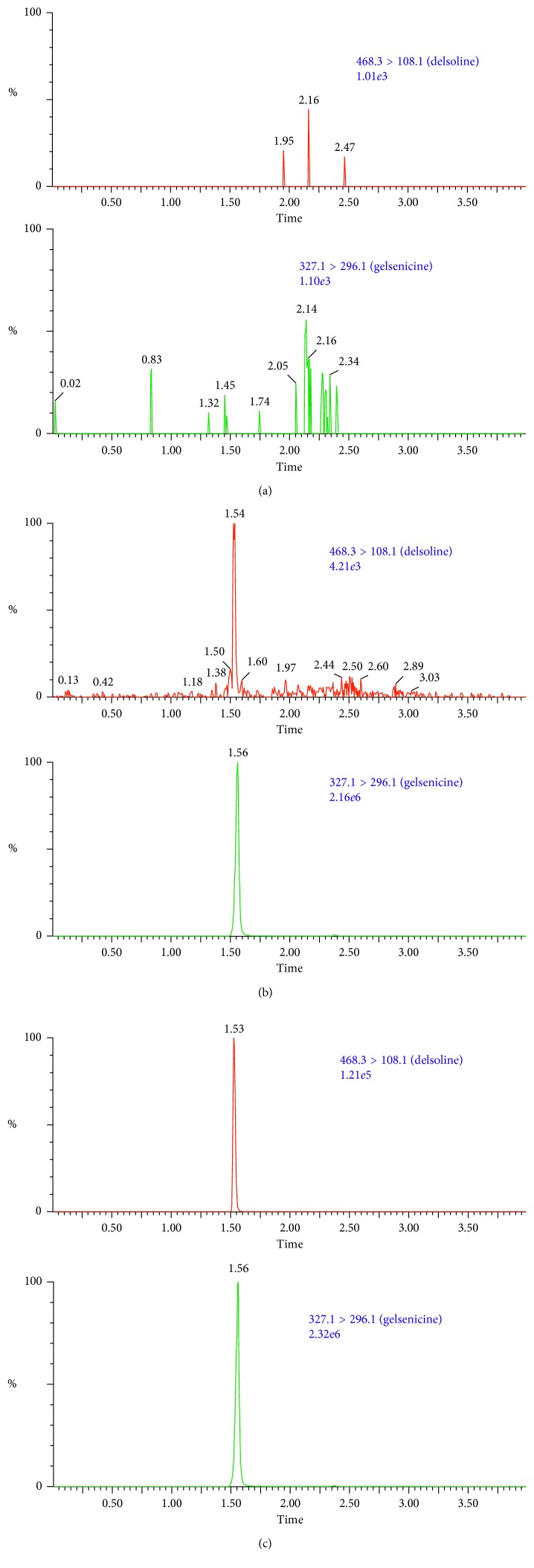
Ultrahigh performance liquid chromatography-mass spectrometry of delsoline and gelsenicine (internal standard) in mouse blood: (a) blank blood; (b) blank blood spiked with delsoline (0.1 ng/mL) and internal standard (20 ng/mL); (c) a mouse blood sample after intragastric administration.

The retention times of delsoline and internal standards were 1.53 and 1.56 min, respectively (Figure 2). No impurities or endogenous substances that would interfere with the test could be identified, indicating that this method had good selectivity.

The equation for the standard curve of delsoline is *Y* = 0.0018*C* + 0.0017, *r*^2^ = 0.9997, where *Y* represents the ratio of the peak areas of delsoline to the internal standard and *C* represents the concentration of delsoline in the blood. The lower limit of quantitation of delsoline in mouse blood was 0.1 ng/mL with the signal-to-noise ratio of 10, and the minimum detection limit was 0.04 ng/mL with the signal-to-noise ratio of 3.

The intraday precision RSD was below 14%, and the interday precision RSD was below 15% (Table 1). The accuracy ranged between 94.3% and 110.1%, the average recovery rate was above 90.8%, and the matrix effect ranged between 97.0% and 102.5%. The above results met the requirements of the pharmacokinetic study of delsoline (acceptance criteria; intraday and interday accuracy: ±15% of nominal concentrations, except ±20% at LLOQ; and intraday and interday precision: ±15% RSD, except ±20% RSD at LLOQ) [12].

Results of freeze-thaw stability tests (room temperature for 2 h; −20°C for 30 days) showed that the variation and RSD of delsoline were within ±12% and within 15%, respectively, indicating that delsoline had good stability.

### 3.3. Pharmacokinetics Study

**Figure 3:**
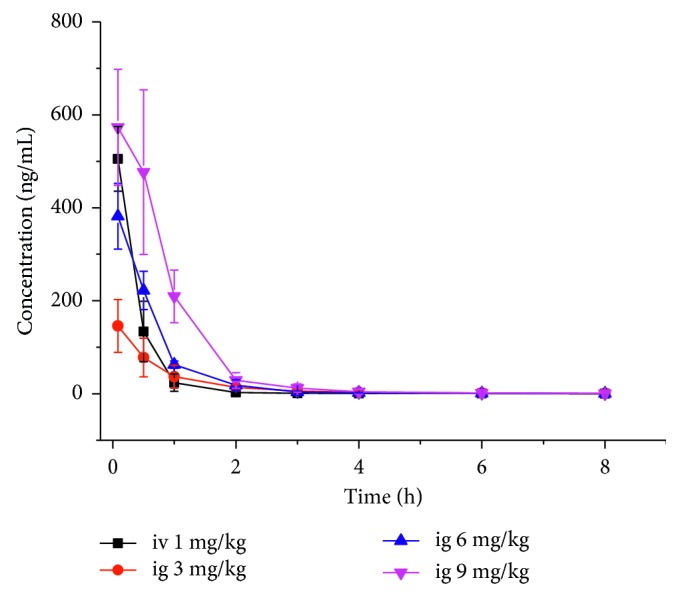
Drug concentration-time curves of delsoline after intragastric administration (3, 6, and 9 mg/kg) and intravenous administration (1 mg/kg) in mice.

The pharmacokinetics of delsoline after intravenous and intragastric administration was studied using the UPLC-MS/MS method. The drug concentration-time curves are shown in Figure 3. The main pharmacokinetic parameters fitted by the noncompartment model are shown in Table 2. The *t*_1/2_ of the intragastric administration (9, 6, and 3 mg/kg) and intravenous administration (1 mg/kg) was 1.3 + 0.5 h, 1.6 + 0.7 h, 1.7 + 0.8 h, and 2.5 + 0.7 h, respectively, indicating that its metabolism was rapid. The absolute bioavailability of delsoline (9, 6, and 3 mg/kg) was 26.2%, 18.8%, and 17.7%, respectively, with an average of 20.9%. The absolute bioavailability of these doses was not significantly different.

## 4. Conclusions

A sensitive, rapid, and selective UPLC-MS/MS method was established for the detection of delsoline in mouse blood. The linear range was 0.1–1000 ng/mL, and the lower limit of quantitation was 0.1 ng/mL. 20 *μ*L blood sample was treated by the direct precipitation method using acetonitrile. We successfully applied this method to study the pharmacokinetics of delsoline after intravenous and intragastric administration, and the bioavailability was determined to be 20.9%.

## Figures and Tables

**Table 1:** Accuracy, precision, matrix effects, and recovery of delsoline in mouse blood.

Concentration (ng/mL)	Accuracy (%)		Precision (RSD, %)		Matrix effect (%)	Recovery (%)
	Intraday	Interday	Intraday	Interday		
0.1	95.6	110.1	13.8	14.2	101.0 ± 6.6	95.3 ± 6.9
0.4	94.3	99.4	5.8	9.4	102.5 ± 6.1	90.8 ± 5.8
180	108.6	101.3	5.1	4.1	99.3 ± 6.9	93.6 ± 1.6
900	105.5	102.5	6.2	6.3	97.0 ± 1.7	94.1 ± 1.3

**Table 2:** Pharmacokinetic parameters after delsoline administration in mice.

Parameters	Unit	ig (9 mg/kg)	ig (6 mg/kg)	ig (3 mg/kg)	iv (1 mg/kg)
AUC_(0–*t*)_	ng/mL*∗*h	570.1 ± 147.5	271.9 ± 25.5	128.4 ± 52.2	241.4 ± 43.9
AUC_(0–*∞*)_	ng/mL*∗*h	571.0 ± 147.5	272.9 ± 25.1	129.9 ± 51.4	242.4 ± 44.0
MRT_(0–*t*)_	H	0.7 ± 0.1	0.7 ± 0.1	1.1 ± 0.2	0.3 ± 0.1
MRT_(0–*∞*)_	H	0.7 ± 0.1	0.7 ± 0.1	1.2 ± 0.3	0.4 ± 0.1
*t*_1/2z_	H	1.3 ± 0.5	1.6 ± 0.7	1.7 ± 0.8	2.5 ± 0.7
CL_z/F_	L/h/kg	16.7 ± 4.4	22.1 ± 1.9	25.6 ± 8.3	4.2 ± 0.8
*V*_z/F_	L/kg	33.1 ± 22.7	51.1 ± 23.0	67.8 ± 50.3	15.4 ± 4.5
*C*_max_	ng/mL	573.2 ± 124.9	381.8 ± 70.8	146.0 ± 56.8	505.3 ± 69.5
Bioavailability		26.2%	18.8%	17.7%	

## Data Availability

The data used to support the findings of this study are available from the corresponding author upon request.
